# Static magnetic field-induced metabolic shifting: coordinated phenylpropanoid induction and antioxidant system regulation in *Calotropis procera* callus culture

**DOI:** 10.1186/s12870-025-07997-3

**Published:** 2026-02-11

**Authors:** Khali M. Saad-Allah, Nour M. Elbana, Sherien E. Sobhy, Elsayed E. Hafez, Asmaa M. Khalifa, Shuhao Huo, Xinjuan Hu, Dina Gad

**Affiliations:** 1 Botany Department, Faculty of Science, Tanta University, Tanta, 31527 Egypt; 2 Plant Protection and Bimolecular Diagnosis Department, Arid Lands Cultivation Research Institute, City of Scientific Research and Technological Applications, New Borg El-Arab, Alexandria, 21934 Egypt; 3 Botany and Microbiology Department, Faculty of Science, Al-Azhar University (Girls Branch), Cairo, 71524 Egypt; 4 School of Food and Biological Engineering, Jiangsu University, Zhenjiang, 212013 China; 5 Botany and Microbiology Department, Faculty of Science, Menoufia University, Shebin EL-Koum, 32511 Egypt

**Keywords:** In vitro culture, Magnetic elicitation, Metabolomics, Redox homeostasis, And gene regulation

## Abstract

**Background:**

*Calotropis procera* is a medicinally significant plant valued for its diverse bioactive pharmacological compounds. Environmental stimuli, such as static magnetic field (SMF), can act as potent elicitors, altering its metabolic pathways. This study investigates the impact of SMF exposure (150 mT) for 0, 1, 2, or 3 h on primary and secondary metabolite components, antioxidant responses, and gene expression of *C. procera* callus cultures.

**Results:**

SMF induced significant, time-dependent metabolic changes. Soluble sugars increased 1.6-fold after 3 h, while soluble proteins declined to 0.47-fold of controls. Phenylpropanoid biosynthesis was markedly enhanced, with phenolics and flavonoids increasing 7.5- and 3.2-fold, respectively. HPLC analysis revealed a coordinated upregulation of phenolic and flavonoid compounds. Kaempferol and ellagic acid showed a 115% increase, while gallic acid and quinic acid derivative increased by over 116%. Conversely, cardiac glycosides and saponins were suppressed. Concurrently, SMF exposure triggered ROS, with levels of O_2_^−•^, H_2_O_2_, OH^•^, and MDA increasing by 462, 117, 160, and 233%, respectively. However, the antioxidant capacity significantly improved, showing 6.91 and 25.93% increases in AsA and GSH levels, alongside 2.32- and 0.30-fold increases in DPPH^•^ scavenging and total antioxidant activity. CAT, POD, and SOD activities declined, while GR activity increased. Gene expression analysis revealed profound upregulation of phenylpropanoid pathway enzymes, particularly PAL (549.89-fold), CHI (100.60-fold), and F3H (50.90-fold).

**Conclusions:**

These results demonstrated that SMF elicited coordinated metabolic reprogramming in *C. procera*, enhancing non-enzymatic antioxidants and phenylpropanoid biosynthesis while suppressing steroidal pathways and enzymatic antioxidant activity, highlighting its potential as a biophysical tool for metabolic engineering.

**Supplementary Information:**

The online version contains supplementary material available at 10.1186/s12870-025-07997-3.

## Background

In recent years, the study of synergistic interactions in phytomedicine has gained significant attention as a promising approach to drug discovery and development. Plants produce complex mixtures of bioactive compounds that often exhibit greater therapeutic effects when combined than when isolated, a phenomenon known as phytochemical synergy [1]. *Calotropis procera* (Ait.) R. Br., a member of the Apocynaceae, is a remarkable medicinal plant known by various names, including Sodom apple, Giant milkweed, and Aak. It thrives in tropical and subtropical regions, particularly in coastal areas of Iran, North Africa, and Southeast Asia [2]. Despite its inherent toxicity, *C. procera* has been widely used in traditional medicine, owing to its rich phytochemical composition. Phytochemical analyses revealed that *C. procera* contains bioactive compounds such as cardenolides, flavonoids, sterols, triterpenoids, and alkaloids, which contribute to its diverse therapeutic properties [3]. These compounds exhibit a broad spectrum of pharmacological activities, including antimicrobial, anti-inflammatory, antioxidant, hepatoprotective, and wound-healing effects [4]. Notably, cardenolides, primarily found in the latex, are crucial in treating congestive heart failure, while flavonoids and their glycosides, abundant in the leaves, contribute to their antioxidant and anti-inflammatory potential [5]. Additionally, terpenoids and oxypregnane glycosides, isolated from the flowers and root bark, further enhance its medicinal value [3].

Recent advances in plant biotechnology have demonstrated that various strategies can significantly enhance the production of valuable secondary metabolites in medicinal plant cell cultures. These approaches include careful selection of high-yielding cell lines, cell immobilization techniques, optimization of culture medium composition, and application of various physical and chemical elicitors [6]. Among these strategies, the application of magnetic fields (MF) has emerged as a particularly promising yet not fully understood technique for stimulating secondary metabolite production. As an unavoidable environmental factor, MF has been shown to influence various growth and developmental processes in living organisms [7]. The proposed mechanisms of MF action include induction of paramagnetic properties in certain cellular molecules, alteration of cell membrane electrical characteristics and permeability, modulation of free radical activity, changes in enzymatic activity within biochemical pathways, and regulation of membrane ionic transport systems [7].

Studies by Abhary and Akhkha [8] have systematically investigated MF effects on plant physiology, revealing significant improvements in seed vigor, vegetative growth parameters, and ultimate crop yield. Their work suggests that MF exposure activates specific molecular pathways that enhance nutrient uptake efficiency and photosynthetic performance. These findings are particularly relevant for medicinal plant cultivation, where secondary metabolite content directly determines therapeutic value. Furthermore, the development of species-specific MF exposure protocols presents a promising frontier in agricultural bioengineering. Current evidence indicates that optimal MF parameters (intensity, duration, and frequency) vary significantly between plant species, likely due to differences in their cellular composition and metabolic pathways [9]. For instance, Alam et al. [10] demonstrated that 75 mT static MF exposure for 45 min increased artemisinin production in *Artemisia annua* by 66%.

Mechanistic studies suggest that MF exposure induces controlled oxidative stress through ROS generation, which subsequently activates plant defense mechanisms and secondary metabolite biosynthesis [11]. This controlled stress response appears to upregulate key enzymes in secondary metabolic pathways while maintaining cellular viability. Recent proteomic analyses have identified MF-induced changes in the expression of chalcone synthase, phenylalanine ammonia-lyase, and other critical enzymes in phenolic compound synthesis [12]. The ability of MF to modulate these fundamental cellular processes without the need for chemical additives makes it an attractive option for improving medicinal plant cell culture systems. To the best of our knowledge, the current literature contains no reported investigations examining the influence of static magnetic field (SMF) exposure on the metabolic processes of *Calotropis procera* callus cultures. This represents a significant gap in our understanding of biophysical elicitation strategies for this medicinally important species. The present study, therefore, seeks to address this knowledge deficit through a comprehensive evaluation of SMF effects on *C. procera* callus culture, with a particular focus on primary and secondary metabolic profiles, changes in antioxidant capacity, and the molecular approaches investigating the differential expression patterns of key genes involved in phenolic and flavonoid biosynthetic pathways, thereby elucidating potential mechanisms underlying any observed metabolic changes. This multidimensional investigation paves the way to establish foundational knowledge regarding SMF applications in *C. procera* biotechnology while contributing to the understanding of magneto-biology in plant systems. The findings may facilitate the development of novel protocols for enhanced production of valuable bioactive compounds from this important medicinal species.

## Methods

### Plant material and callus development

Fresh and healthy aerial branches (including leaves and stems) of *Calotropis procera* (Ait.) R. Br. were collected in August 2024 from a natural wild population located along the Cairo–Suez road, Egypt (30°09′58″ N, 31°83′67″ E). The collection of plant material was conducted in full compliance with Egyptian national legislation for the protection of wild plants. Although that there are no specific licenses were required for the field studies, formal permission for collection was obtained from the Egyptian Environmental Affairs Agency (EEAA) prior to fieldwork. The collection was performed under the guidelines and rules of Tanta University. The formal identification of the plant specimens was performed by Prof. Kamal H. Shaltout, Professor of Plant Ecology and Taxonomy in the Botany Department, Faculty of Science, Tanta University. A voucher specimen (accession number: TAN56771) was prepared and deposited in the Herbarium of Tanta University (TAN) for future reference.

Plant material was transported on ice to the Genetics Laboratory, Faculty of Science, Tanta University, Egypt. The samples were thoroughly washed under running tap water and subsequently rinsed three times with sterile distilled water. Leaves were cut into approximately 1 × 1 cm segments under aseptic conditions. Surface sterilization was performed by immersing the explants in 70% (v/v) ethanol for 1 min, followed by treatment with 1.5% (v/v) sodium hypochlorite solution for 10 min. Finally, the segments were rinsed three times with autoclaved distilled water to remove residual disinfection liquid. For callus induction, sterilized leaf explants were cultured on solid MS medium [13], supplemented with 1.0 mg L^− 1^ benzylaminopurine (BAP), 0.5 mg L^− 1^ 2,4-dichlorophenoxyacetic acid (2,4-D), 30 g L^− 1^ sucrose, and 10 g L^− 1^ agar [14]. The pH of the medium was adjusted to 5.8 before autoclaving at 121 °C and 1.1 atm for 20 min. Cultures were maintained in darkness at 25 ± 2 °C. Callus formation was observed after approximately 30 days of incubation.

Following callus induction, uniformly growing *C. procera* callus cultures were subjected to a static magnetic field (SMF) exposure protocol using a custom-built electromagnetic coil system at the Physics Laboratory, Faculty of Science, Tanta University. The exposure apparatus consisted of a solenoid coil, comprising 17,000 turns of copper wire wound on a laminated wooden core (30 cm length, 17 cm inner radius), powered by a Global Dual Power Supply (Model 3521) generating a 150 millitesla (mT) field. The magnetic field intensity was calibrated and verified for homogeneity using gauss meter (F.W. Bell, Model 5080), measured across the central exposure zone (8–22 cm vertically from the coil base). Glass jars containing callus cultures were placed within this uniform field region and subjected to SMF for durations of 0, 1, 2, or 3 h per day over three successive days, while maintaining a constant temperature of 25 ± 1 °C. Control samples (0 h) were placed outside the influence of the generated SMF, exposed only to the Earth’s natural magnetic field. Following the treatment period, all callus cultures were returned to standard growth conditions. After one week of the final SMF exposure, these callus cultures were harvested, immediately frozen in liquid nitrogen, and stored at -80 °C for biochemical and molecular analyses. The experimental design included three replicates per treatment.

### Quantification of primary metabolites

The extraction of soluble sugars and soluble proteins from *C. procera* callus cultures was conducted using a standardized protocol. Briefly, 0.5 g of each sample was homogenized in a pre-chilled mortar and pestle with cold borate buffer (pH 8.0) as prescribed by Karppinen et al. [15]. The resulting homogenate was subjected to centrifugation at 10,000 rpm for 15 min in a refrigerated centrifuge to separate the soluble fraction. The supernatant obtained was subsequently utilized for the quantification of soluble sugars and proteins.

For the determination of total soluble sugars (TSS), a 0.5 ml aliquot of the extract was reacted with 0.5 mL of 5% phenol and 1.0 mL of H_2_SO_4_. The mixture was incubated at 60 °C for 20 min in a water bath. After cooling, the absorbance was measured at 490 nm. The TSS concentration was quantified using a standard curve generated with glucose as a reference sugar [16]. Total soluble protein (TSP) content was assessed by mixing 0.5 mL of the borate buffer extract with 2.5 mL of Coomassie Brilliant Blue G-250 reagent. Following a 10-min incubation period, the absorbance was recorded at 595 nm. Protein concentration was determined based on a standard curve prepared using bovine serum albumin (BSA) as the calibration standard [17].

For quantifying free amino acids (FAA), an ethanolic extraction procedure was employed. A sample of 0.5 g of fresh *C. procera* callus tissue was homogenized in 10 mL of 95% ethanol. A 0.1 mL aliquot of the resulting extract was then combined with 1.9 mL of acidified ninhydrin reagent and heated in a boiling water bath for 12 min. After cooling, the absorbance was measured at 570 nm. The FAA concentration was calculated based on a standard calibration curve constructed using glycine as the reference compound [18].

### Quantitative analysis of secondary metabolites

The ethanolic extracts of *C. procera* callus samples were analyzed for various secondary metabolites using standardized spectrophotometric methods. The alkaloid content was determined following the protocol of Shamsa et al. [19]. The ethanolic extract was reacted with bromocresol green (BCG) to form a yellow alkaloid-BCG complex, which was then extracted into chloroform. The absorbance of the chloroform layer was measured at 470 nm, and the alkaloid concentration was quantified using atropine as a reference standard. Saponins were estimated according to the method of Hiai et al. [20]. 0.5 mL of ethanolic extract was mixed with 0.5 mL of 8% vanillin in ethanol, chilled in an ice bath, and then treated with 5 mL of 72% H_2_SO_4_. The mixture was heated at 60 °C for 10 min, cooled, and the absorbance was recorded at 544 nm. A standard curve generated from cholesterol was used for quantification.

Cardiac glycosides were assessed using a modified Baljet’s reagent method [21]. A 0.5 mL aliquot of the extract or the standard digoxin was combined with 2.5 mL of freshly prepared Baljet’s reagent (1% picric acid and 10% NaOH in a 95:5 ratio). After 60 min incubation, the absorbance was measured at 495 nm. Terpenoids were quantified by the approach of Ghorai et al. [22]. A 200 µL aliquot of the extract was mixed with 1.5 mL chloroform, followed by the addition of 100 µL H_2_SO_4_. The mixture was incubated in the dark for 2 h, after which the supernatant was decanted, and the pellets were dissolved in 1.5 mL of 95% methanol. Absorbance was measured at 538 nm, and the terpenoid content was calculated using a linalool-based standard curve.

Flavonoids were estimated using the method of Chang et al. [23]. A 0.5 ml ethanolic extract was mixed with 1.5 mL of 95% ethanol, 0.1 mL of 10% AlCl_3_, 0.1 mL of 1 M K-acetate, and 2.8 mL of distilled water. After 30 min of incubation, absorbance was measured at 415 nm. Quercetin was used as the reference standard, with a blank prepared by replacing AlCl_3_ with distilled water. Total phenolics were determined following the Folin-Ciocalteu method [24]. A 1 mL extract was mixed with 0.1 mL Folin-Ciocalteu reagent and 1 mL of 20% Na_2_CO_3_, then diluted to 5 mL with distilled water. After 30 min, absorbance was recorded at 650 nm, and gallic acid was used to produce the standard calibration curve.

### HPLC analysis of phenols and flavonoids

Phenolic and flavonoid constituents in ethanolic extracts of *C. procera* callus cultures were identified using high-performance liquid chromatography (HPLC). The analysis was performed on an Agilent 1260 series system equipped with a Zorbax Eclipse Plus C8 column (4.6 × 250 mm, 5 μm). Separation was achieved with a gradient elution of water (mobile phase A) and 0.05% trifluoroacetic acid in acetonitrile (mobile phase B) at a flow rate of 1.0 mL/min. The column temperature was maintained at 40 °C, and detection was carried out at 280 nm with a 5 µL injection volume. The gradient program was optimized as follows: 0–1 min, 82% A; 1–11 min, 75% A; 11–18 min, 60% A; 18–22 min, 82% A [25].

### Oxidative stress-related markers

Malondialdehyde (MDA) content, a marker of lipid peroxidation, was determined using the thiobarbituric acid (TBA) assay according to Heath and Packer [26]. *C. procera* callus samples (500 mg) were homogenized in 10% trichloroacetic acid (TCA) and centrifuged at 6000 rpm for 20 min. The supernatant was boiled with 0.67% TBA in 10% TCA at 100 °C for 30 min. The absorbance was measured at 532 nm and 600 nm for turbidity correction. MDA concentration (µmol g^− 1^ FM) was calculated using an extinction coefficient of 155 mM^− 1^ cm^− 1^.

The hydrogen peroxide (H_2_O_2_) content was assayed following Velikova et al. [27] approach. Callus tissue (100 mg) was extracted in 5 mL of 0.1% TCA and centrifuged at 6000 rpm for 15 min. From the supernatant, a 0.5 mL aliquot was reacted with 0.5 mL of 10 mM phosphate buffer (pH 7.0) and 1 mL of 1 M potassium iodide. The absorbance was read at 390 nm, and the H_2_O_2_ content (nmol g^− 1^ FM) was calculated using an extinction coefficient of 0.28 µM^− 1^ cm^− 1^.

The superoxide anion (O_2_^−•^) production rate was assayed using a modified hydroxylamine oxidation method [28]. Fresh tissue samples (200 mg) were homogenized in 1 mL phosphate buffer (50 mM, pH 7.8) and centrifuged at 7000 rpm for 10 min. A 0.5 mL supernatant aliquot was reacted with 0.5 mL of phosphate buffer and 0.1 mL of 10 mM hydroxylamine hydrochloride for 1 h at 25 °C. Subsequently, 1 mL of sulfanilamide (17 mM) and 1 mL of α-naphthylamine (7 mM) were added. After a 20-min incubation, the absorbance was read at 530 nm. The production rate of O_2_^−•^ (µmol min^− 1^ g^− 1^ FM) was calculated from a standard curve generated with sodium nitrite.

Hydroxyl radical (OH^•^) content was assayed based on the method of Halliwell et al. [29]. Callus samples (500 g) were homogenized in 10 mM phosphate buffer saline (pH 7.4) containing 15 mM 2-deoxy-D-ribose and centrifuged at 8000 rpm for 15 min. The supernatant was incubated at 37 °C for 2 h. Subsequently, 0.7 mL of extract was added to a reaction mixture of 0.5% TBA in glacial acetic acid and heated at 100 °C for 30 min. The absorbance of the resulting solution was measured at 532 nm. The OH^•^ content (nmol g^− 1^ FM) was calculated based on an extinction coefficient of 155 mM^− 1^ cm^− 1^.

### Antioxidant compounds and antioxidant potential

The ascorbic acid content in *C. procera* callus tissues was quantified following the method described by Oser [30]. Briefly, 0.5 g of fresh callus tissue was homogenized in 5 mL of 5% (w/v) sulfosalicylic acid and centrifuged at 10,000 rpm for 10 min. The reaction mixture consisted of 2 mL of 2% Na_2_MoO_4_.2H_2_O, 2 mL of 0.15 N H_2_SO_4_, 1 mL of 1.5 mM Na_2_HPO_4_, and 1 mL of the supernatant. After incubation at 60 °C for 40 min, the mixture was cooled, centrifuged at 5,000 rpm for 10 min, and the absorbance was measured at 660 nm. The ascorbic acid concentration was determined using a standard calibration curve prepared with pure ascorbic acid.

The reduced glutathione (GSH) content was determined according to the method of Andersen [31]. A 0.5 g sample was extracted in 3 mL of 5% sulfosalicylic acid and centrifuged. A 0.5 mL supernatant was mixed with 100 mM K-phosphate buffer (pH 7.0), 0.5 mM EDTA, and 3 mM 5,5′-dithiobis (2-nitrobenzoic acid) (DTNB). The absorbance was recorded at 412 nm, and the GSH concentration (µg g^− 1^ FM) was calculated using a GSH standard curve.

The free radical scavenging activity of *C. procera* ethanolic extracts was evaluated using the 2,2-diphenyl-1-picrylhydrazyl (DPPH) assay [32] with slight modifications. A 0.1 mL ethanolic extract was mixed with 3.9 mL of a 0.03 g l^− 1^ DPPH methanolic solution. The mixture was vortexed, incubated in the dark for 60 min, and the absorbance was measured at 517 nm. Blank ethanol instead of the extract was used for baseline correction. The DPPH radical scavenging activity (%) was calculated as follows:

$$\begin{aligned}\mathrm{D}\mathrm{P}\mathrm{P}\mathrm{H}\:\mathrm{s}\mathrm{c}&\mathrm{a}\mathrm{v}\mathrm{e}\mathrm{n}\mathrm{g}\mathrm{i}\mathrm{n}\mathrm{g}\:\mathrm{a}\mathrm{c}\mathrm{t}\mathrm{i}\mathrm{v}\mathrm{i}\mathrm{t}\mathrm{y}\:\left(\%\right)\\&=\left(\frac{\mathrm{A}0-\mathrm{A}\mathrm{s}}{\mathrm{A}0}\right)\times100\end{aligned}$$

Where A0 is the blank absorbance and As is the sample absorbance.

The total antioxidant capacity was assessed using the phosphomolybdate method [33]. A 300 µL extract was mixed with 3 mL of phosphomolybdate reagent (0.6 M H_2_SO_4_, 28 mM Na_3_PO_4_, and 4 mM (NH4)_6_Mo_7_O_24_·4H_2_O). The mixture was incubated at 95 °C for 90 min, cooled, and the absorbance was measured at 765 nm. A blank without the sample was processed similarly. The total antioxidant activity was expressed in µg ml^− 1^ of ascorbic acid equivalents (AAE) using a standard calibration curve.

### Extraction and determination of antioxidant enzyme activities

Fresh callus samples (500 mg) were homogenized in 8 mL of ice-cold 50 mM Tris-HCl buffer (pH 6.8) containing 50 mg PVP, 10 mM DTT, and 0.1 mM EDTA. The homogenate was centrifuged at 10,000 rpm for 15 min at 4 °C, and the resulting supernatant was used for subsequent enzyme assays.

Superoxide dismutase (SOD) activity was determined according to the photochemical nitroblue tetrazolium (NBT) reduction method [34]. The reaction mixture (1 mL) contained 50 mM K-phosphate buffer (pH 7.8), 13 mM L-methionine, 75 µM NBT, 2 µM riboflavin, and 0.1% Triton X-100. The reaction was initiated by illuminating the samples for 15 min under fluorescent light (25 W) in an aluminum foil-lined chamber. Absorbance was measured at 560 nm, and SOD activity was calculated using the extinction coefficient (21.1 mM^− 1^cm^− 1^).

Catalase (CAT) activity was assayed by monitoring H_2_O_2_ decomposition at 240 nm [35]. The reaction mixture (3 mL) consisted of 50 mM K-phosphate buffer (pH 7.0) and 10.5 mM H_2_O_2_. The reaction was initiated by adding 100 µL of enzyme extract, and the decrease in absorbance was recorded for 1 min. Enzyme activity was calculated using the molar extinction coefficient of H_2_O_2_ (43.6 mM^− 1^ cm^− 1^).

Peroxidase (POD) activity was measured according to Nakano and Asada [36] by monitoring the oxidation of guaiacol at 470 nm. The reaction mixture (2 mL) contained 50 mM K-phosphate buffer (pH 7.0), 2 mM H_2_O_2_, and 2.7 mM guaiacol. The reaction was initiated with 100 µL of enzyme extract, and the increase in absorbance due to tetraguaiacol formation was recorded. POD activity was calculated using the extinction coefficient of 26.6 mM^− 1^ cm^− 1^.

Polyphenol oxidase (PPO) activity was determined using pyrogallol as substrate [37]. The reaction mixture (2 mL of 100 mM K-phosphate buffer, pH 6.0, and 1 mL of 20 mM pyrogallol) was mixed with 500 µL enzyme extract. After 5 min incubation at 25 °C, the reaction was terminated with 1 mL of 2.5 N H_2_SO_4_. The absorbance of purpurogallin was measured at 420 nm, and activity was calculated using 26.4 mM^− 1^ cm^− 1^ as an extinction coefficient.

Glutathione reductase (GR) activity was assayed by monitoring NADPH oxidation at 340 nm [38]. The reaction mixture (1 mL) contained 100 mM Tris-HCl (pH 7.8), 21 mM EDTA, 0.5 mM oxidized glutathione, and 5 µM NADPH. The reaction was initiated with NADPH, and the decrease in absorbance was recorded. Activity was calculated using the NADPH extinction coefficient (6.2 mM^− 1^ cm^− 1^).

Ascorbate peroxidase (APX) activity was determined by measuring ascorbate oxidation at 290 nm [36]. The reaction mixture (2 mL) contained 50 mM K-phosphate buffer (pH 7.0), 0.2 mM EDTA, 0.5 mM ascorbate, and 0.25 mM H_2_O_2_. The reaction was initiated with the enzyme extract, and the decrease in absorbance was recorded. Activity was calculated using the ascorbate extinction coefficient (2.8 mM^− 1^ cm^− 1^).

### Quantitative real-time PCR (qRT-PCR) analysis

Total RNA was isolated from *C. procera* callus samples using the RNeasy Mini Kit (Qiagen, Germany) following the manufacturer’s protocol. RNA purity was confirmed spectrophotometrically (A260/A280 ratio ≥ 1.8). First-strand cDNA synthesis was performed in 20 µL reactions using a PTC-100™ thermal cycler (MJ Research, Inc., PTC-100™ Programmable thermal controller, USA) with the following parameters: 42 °C for 60 min (reverse transcription) followed by 80 °C for 15 min (enzyme inactivation). Quantitative PCR amplifications were performed in triplicate 25 µL reactions containing SYBR Green PCR Master Mix (Fermentas) and gene-specific primers (Table 1) targeting phenylpropanoid pathway genes (HQT, HCT, C3H, DFR, CHS, CHI, F3H, and PAL). Reactions were carried out in a Rotor-Gene 6000 system (QIAGEN, ABI System, USA) with data acquisition during the extension phase. Thermal cycling conditions followed the protocol established by Sobhy et al. [39], with melt curve analysis confirming amplification specificity. Gene expression levels were normalized to the endogenous β-actin reference gene and quantified using the comparative ^2–^∆∆CT method [40].

**Table 1:** qRT-PCR sequence of phenylpropanoid pathway primers used in this investigation

Gene name	Abbreviation	Direction	Sequences 5 ـــــــــ 3
Chalcone synthase	CHS	F	AGGCTAACAGAGGAGGGTA
		R	CCAATTTACCGGCTTTCT
Chalcone isomerase	CHI	F	TGGTGGCCTAGACAACGATGAGTT
		R	TCACACTCCCAACTTGGTTTCCCT
Flavanone 3-hydroxylase	F3H	F	CCAAGGCATGTGTGGATATGGACC
		R	CCTGGATCAGTATGTCGTTCAGCC
*p*-coumarate 3-hydroxylase	C3H	F	TTGGTGGCTACGACATTCCTAAGG
		R	GGTCTGAACTCCAATGGGTTATTCC
Dihydrofavonol 4-reductase	DFR	F	TCACAGGAGCAGCTGGATTTATCG
		R	TCAGGATCACGAACAGTAGCATGG
Hydroxycinnamoyl Co A: shikimate hydroxycinnamoyl transferase	HCT	F R	TCTCCAACCCCTTTTAACGAACC
			CAACTTGTCCTTCTACCACAGGGAA
Hydroxycinnamoyl CoA quinate hydroxycinnamoyl transferase	HQT	F	CCCAATGGCTGGAAGATTAGCTA
		R	CATGAATCACTTTCAGCCTCAACAA
Phenylalanine ammonia lyase	PAL	F R	ACGGGTTGCCATCTAATCTGACA
			CGAGCAATAAGAAGCCATCGCAAT
Reference gene	ß-actin	F	GTGGGCCGCTCTAGGCACCAA
		R	CTCTTTGATGTCACGCACGATTTC

### Statistical analysis

All experimental data are presented as mean values ± standard deviation (SD) derived from a minimum of three independent replicates. Statistical comparisons were performed using one-way analysis of variance (ANOVA) implemented in CoStat software (version 6.311, CoHort). Multiple post-hoc comparisons were conducted using Duncan’s test at a significance level of *P* < 0.05. Results were considered statistically significant when *P* < 0.05 unless otherwise noted. Correlation analyses were visualized through Pearson correlation matrix heatmap generated using GraphPad Prism software (v. 8.3.0).

## Results

### Primary metabolite accumulation in *C. procera* callus cultures

**Fig. 1:**
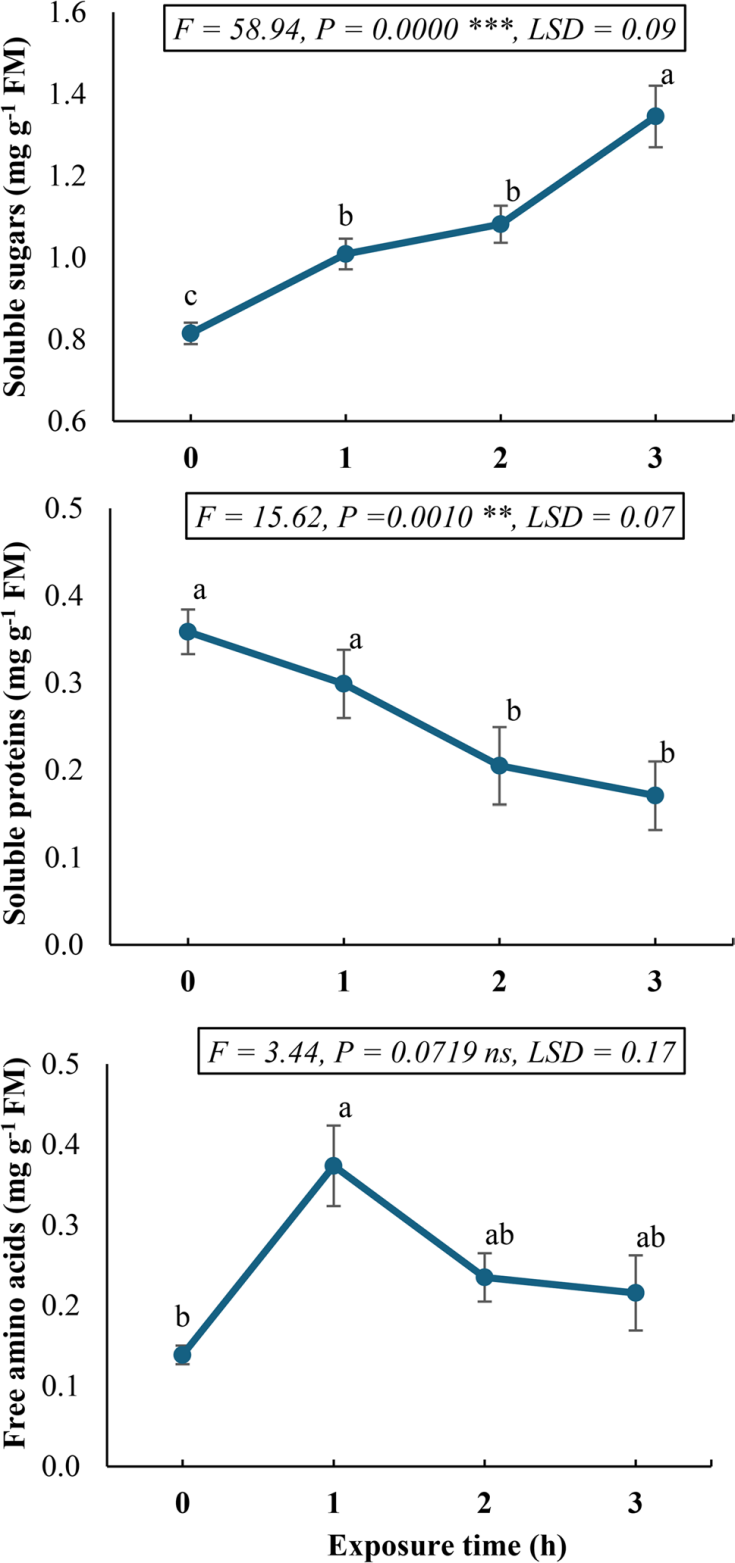
Primary metabolite profile (sugars, proteins, and amino acids) in *Calotropis procera* callus culture following exposure to a 150 mT static magnetic field for varying durations. Different letters indicate statistically significant differences (*P* < 0.05) as determined by Duncan post-hoc test

The experimental results demonstrated significant time-dependent variations in primary metabolite accumulation in *Calotropis procera* callus following static magnetic field (SMF) exposure (Fig. 1). Total soluble sugar content significantly (*P* < 0.05) exhibited progressive enhancement, peaking at 3 h post-exposure with a 1.6-fold increase relative to untreated controls. Conversely, total soluble protein concentration displayed a significant inverse relationship with exposure time, showing maximal reduction (0.47-fold of the control value) after 3 h of SMF treatment. Free amino acid levels demonstrated a non-significant (*P* > 0.05) biphasic regulation, with an initial 2.6-fold increase at 1 h exposure followed by a gradual decline, though remaining significantly elevated (0.8-fold) above control levels after 3 h of treatment.

### Secondary metabolite accumulation in *C. procera* callus cultures

**Fig. 2:**
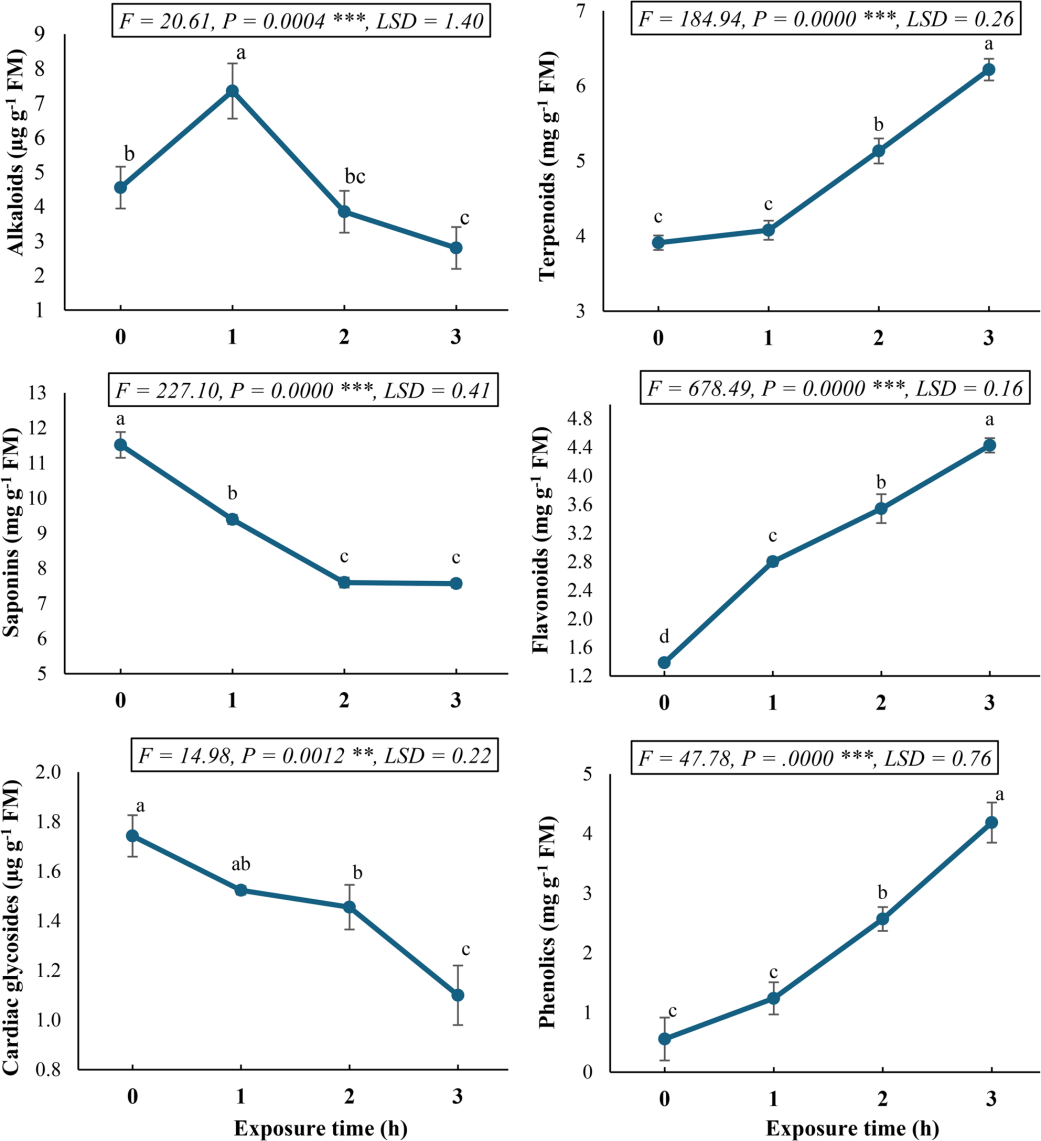
Secondary metabolite profile (alkaloids, saponins, glycosides, terpenoids, flavonoids, and phenolics) in *Calotropis procera* callus culture following exposure to a 150 mT static magnetic field for varying durations. Different letters indicate statistically significant differences (*P* < 0.05) as determined by Duncan post-hoc test

The static magnetic field (SMF) exposure duration significantly (*P* < 0.05) modulated the biosynthesis of secondary metabolites, as a key player in the therapeutic potential, in *C. procera* callus cultures (Fig. 2). Time-course analysis revealed differential accumulation patterns among various metabolite classes. Phenylpropanoid-derived compounds exhibited particularly pronounced responses, with flavonoid and phenolic contents demonstrating 3.2- and 7.5-fold increases, respectively, after 3 h of SMF exposure. Terpenoid accumulation showed more moderate enhancement, reaching 1.6-fold of control levels at the 3-h timepoint.

Contrasting responses were observed for steroidal compounds. Cardiac glycosides displayed progressive decline, attaining maximal reduction (0.6-fold of control) after 3 h of SMF exposure. Saponin content followed a similar but more rapid decline, reaching its nadir (0.65-fold of control) after 2 h of SMF treatment. Alkaloid metabolism exhibited a biphasic pattern, like that of free amino acids, with an initial 0.61-fold increase after 1 h, followed by progressive decline to 0.34-fold below control levels after 3 h of SMF exposure. These findings demonstrate the selective metabolic reprogramming induced by SMF in *C. procera*, with particularly robust stimulation of phenylpropanoid pathways (flavonoids and phenolics) accompanied by suppression of steroidal metabolites (cardiac glycosides and saponins). The transient alkaloid accumulation suggests potential stress-responsive activation followed by metabolic redirection. The time-dependent nature of these responses indicates dynamic biochemical adaptation to magnetic stimuli in plant cell cultures.

### Phenol and flavonoid profiles of *C. procera* callus cultures as determined by HPLC

The application of a 150 mT SMF induced a pronounced, time-dependent accumulation of phenol and flavonoid constituents in *C. procera* callus cultures. The data demonstrated a clear positive correlation between exposure duration (0–3 h) and the concentration of all identified phenolic and flavonoid compounds (Table 2 and Figure S1). A total of 18 phenolic and flavonoid compounds were identified in *C. procera* callus cultures, irrespective of the duration of SMF exposure. The most prominent response was observed in the flavonol kaempferol, which exhibited the highest concentration across all time intervals. After 3 h of SMF exposure, kaempferol content increased from 2.056 to 4.418 µg g^− 1^ FM, representing a 115% increase. Similarly, the polyphenol ellagic acid showed a substantial increment from 2.87 to 6.165 µg g^− 1^ FM (115% increase) corresponding the relative enhancement of kaempferol. Other compounds with marked increases included the quinic acid derivative (116%), salicylic acid (114%), gallic acid (117%), p-hydroxybenzoic acid (114%), and ferulic acid (83%).

**Table 2:** HPLC analysis of phenolic and flavonoid components in *Calotropis procera* callus after 150 mT SMF exposure

Peak #	RT (min)	Compound Name	Concentration (µg g^− 1^ FM)				Chemical class
			0 h	1 h	2 h	3 h	
1	3.124	Catechol	0.037	0.051	0.068	0.078	Polyphenol
2	4.276	Caffeic acid	0.461	0.566	0.715	0.874	Phenolic acid
3	5.398	Ferulic acid	1.028	1.272	1.563	1.879	Phenolic acid
4	6.564	o-Coumaric acid	0.278	0.363	0.479	0.599	Phenolic acid
5	6.893	Gallic acid	0.507	0.669	0.858	1.098	Phenolic acid
6	7.058	Quercetin	0.647	0.848	1.081	1.399	Flavonol
7	7.115	Quinic acid derivative	1.038	1.353	1.742	2.239	Phenolic acid derivative
8	7.959	Chlorogenic acid	0.489	0.632	0.827	1.058	Phenolic acid
9	8.137	Kaempferol	2.056	2.670	3.449	4.418	Flavonol
10	8.647	Syringic acid	0.379	0.490	0.637	0.815	Phenolic acid
11	9.005	Luteolin	0.312	0.416	0.529	0.672	Flavanone
12	9.477	Myrciacitrin IV	0.278	0.369	0.460	0.590	Flavanone glucoside
13	9.972	p-Hydroxybenzoic acid	1.357	1.761	2.279	2.910	Phenolic acid
14	10.428	Cinnamic acid	0.455	0.599	0.753	0.971	Phenolic acid
15	11.234	Salicylic acid	1.670	2.178	2.808	3.580	Phenolic acid
16	12.354	Ellagic acid	2.87	3.751	4.810	6.165	Polyphenol
17	14.367	Protocatechuic acid	0.369	0.488	0.609	0.799	Phenolic acid
18	19.379	Kaempferol glucoside derivative	0.267	0.340	0.458	0.593	Flavonol glucoside

The analysis further identified compounds of intermediate abundance, including caffeic acid, quercetin, chlorogenic acid, syringic acid, luteolin, cinnamic acid, and protocatechuic acid (0.312–0.647 µg g^− 1^ FM), as well as less abundant constituents, including catechol, o-coumaric acid, myrciacitrin, and kaempferol glucoside derivative (0.037–0.267 µg g⁻¹ FM). Notably, all quantified compounds, regardless of their initial concentration, exhibited a time-dependent increase in accumulation in response to duration of SMF exposure. This coordinated upregulation in phenolic acids, flavonols, and flavanones showed that SMF exposure acted as a potent abiotic elicitor, stimulating the enzymatic pathways of phenylpropanoid metabolism.

### Oxidative stress biomarkers in *C. procera* callus cultures following SMF exposure

**Fig. 3:**
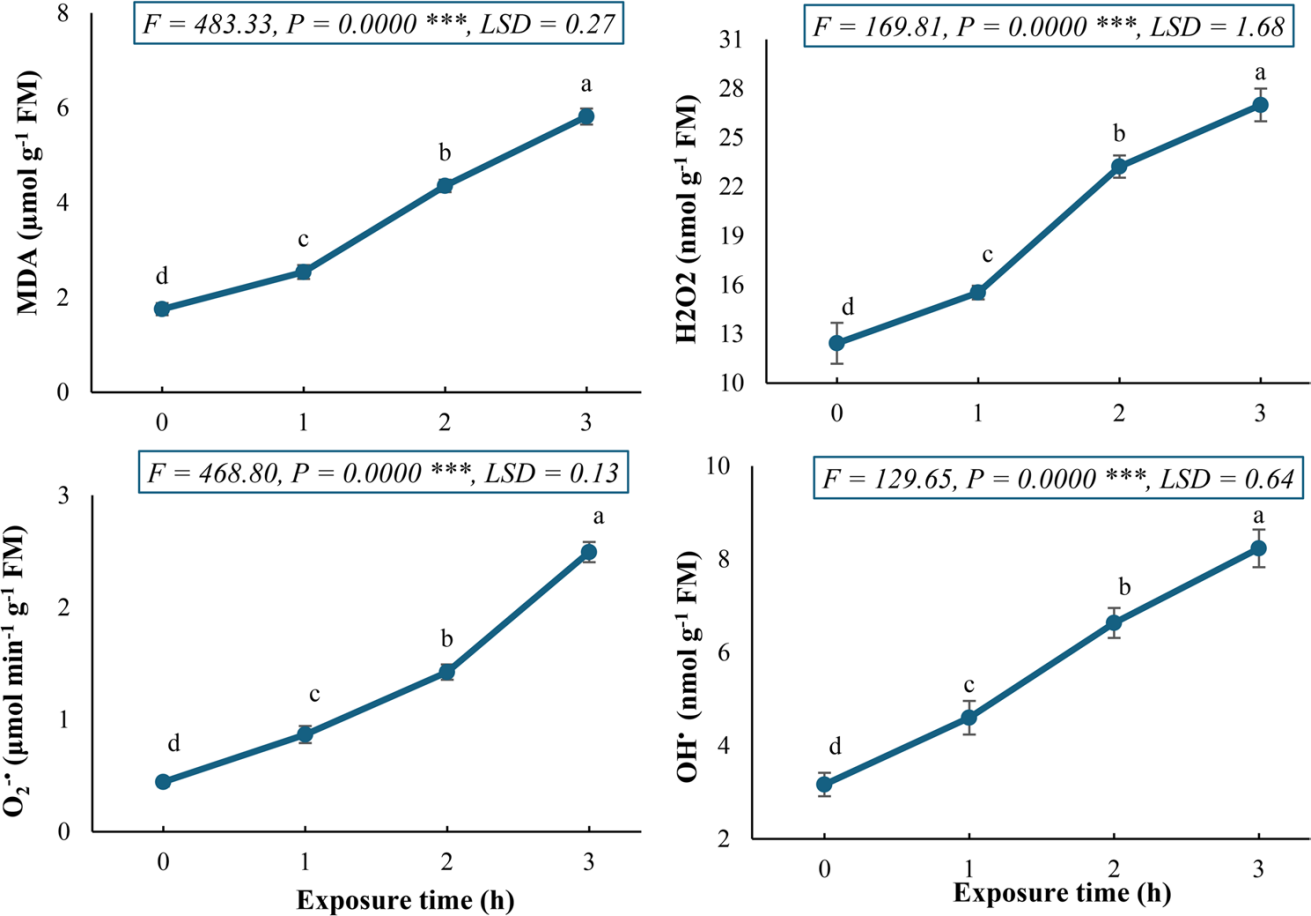
Oxidative stress biomarkers (MDA, O2-•, H2O2, and OH•) in *Calotropis procera* callus culture following exposure to a 150 mT static magnetic field for varying durations. Different letters indicate statistically significant differences (*P* < 0.05) as determined by Duncan post-hoc test

The data presented in Fig. 3 demonstrates a time-dependent induction of oxidative stress in biomarkers (malondialdehyde, hydrogen peroxide, superoxide radical, and hydroxyl radical) in *C. procera* callus cultures exposed to a 150 mT SMF for different time intervals (0–3 h). The data revealed a coordinated and progressive increase in the assessed key biomarkers of oxidative damage. A significant increase in malondialdehyde (MDA) content was observed, rising from 1.75 to 5.82 µmol g^− 1^ FM (233% increase) due to the callus exposure to 150 mT for 3 h, indicating a high level of lipid peroxidation due to severe oxidative damage to cellular membranes.

Concurrently, hydrogen peroxide (H_2_O_2_) levels showed a marked increase from 12.43 to 27.00 nmol g^− 1^ FM (117% increase following the callus exposure to 150 mT for 3 h, suggesting the disruption of cellular redox homeostasis. Likewise, the most marked response was recorded for the superoxide anion (O_2_^−•^), which showed a 462% increase (from 0.44 to 2.49 µmol min^− 1^ g^− 1^ FM) after 3 h of exposure to SMF. This distinct generation of O_2_^−•^ indicates that the SMF stimulates the one-electron reduction of molecular oxygen, generating reactive oxygen species at a significantly high rate. Furthermore, the hydroxyl radical (OH^•^) content increased by 160% (from 3.17 to 8.23 nmol g^− 1^ FM) after 3 h of exposure to SMF. Thus, the generation of ROS (H_2_O_2_, O_2_^−•^, and OH^•^) explains the concomitant rise in membrane damage via lipid peroxidation, as an integrative response to SMF. Collectively, the coordinated and significant increase in these biomarkers confirms that the 150 mT SMF acts as a potent abiotic elicitor, prompting a rigorous oxidative burst that overwhelms the innate antioxidant defense machinery of the callus cells.

### Modulation of antioxidant defense system by SMF in *C. procera* callus cultures

**Fig. 4:**
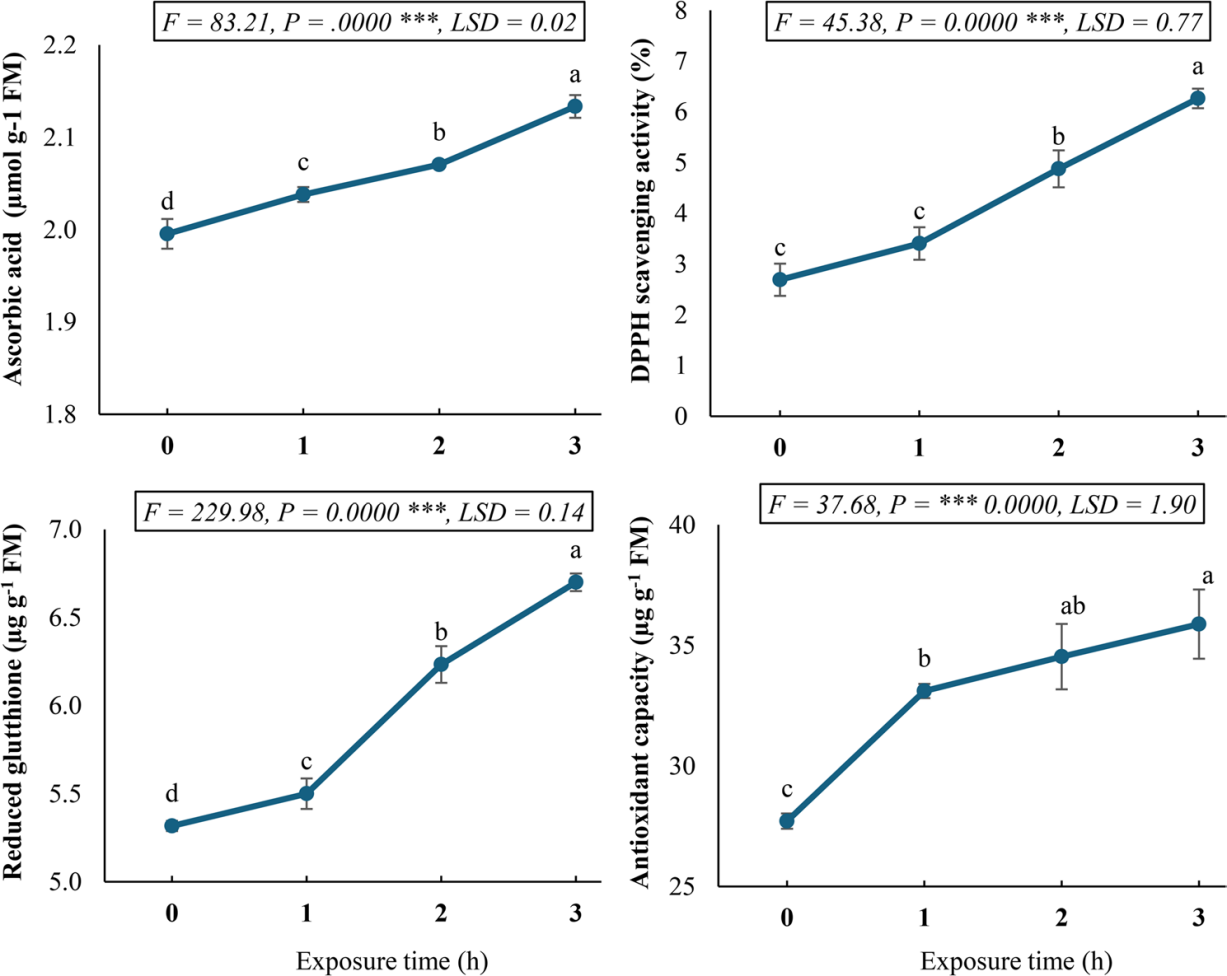
Antioxidant compounds (ascorbic acid, and reduced glutathione) and antioxidant potential (DPPH, and total antioxidant capacity) in *Calotropis procera* callus culture following exposure to a 150 mT static magnetic field for varying durations. Different letters indicate statistically significant differences (*P* < 0.05) as determined by Duncan post-hoc test

The antioxidant capacity of plant tissues is fundamentally determined by their endogenous antioxidant content. Our investigation revealed significant time-based modulation of key non-enzymatic antioxidants in *C. procera* callus cultures exposed to a static magnetic field (SMF) (Fig. 4). Quantitative analysis demonstrated exposure time-dependent accumulation of both ascorbic acid (AsA) and reduced glutathione (GSH), with maximal concentrations achieved after 3 h of SMF treatment. Specifically, AsA content peaked at 2.13 µmol g^− 1^ FM, representing a 6.91% increase over control values, while GSH reached 6.70 µg g^− 1^ FM, corresponding to a 25.93% enhancement relative to untreated cultures.

This upregulation of cellular antioxidants directly correlated with enhanced antioxidant potential. Both DPPH radical scavenging activity and total antioxidant activity, as measured by phosphomolybdenum reduction assay, exhibited progressive enhancement with increasing SMF exposure duration. After 3 h of treatment, cultures demonstrated 2.32-fold greater DPPH radical scavenging activity and 0.30-fold higher total antioxidant capacity compared to control samples. The synchronous increase in antioxidant metabolites and corresponding functional activity suggests coordinated activation of the cellular redox buffering system in response to electromagnetic stimulation. Thus, SMF exposure triggers a time-dependent antioxidant response in *C. procera* callus cultures, with a differential magnitude of response between AsA and GSH accumulation.

### Modulation of antioxidant enzymes by SMF in *C. procera* callus cultures

**Fig. 5:**
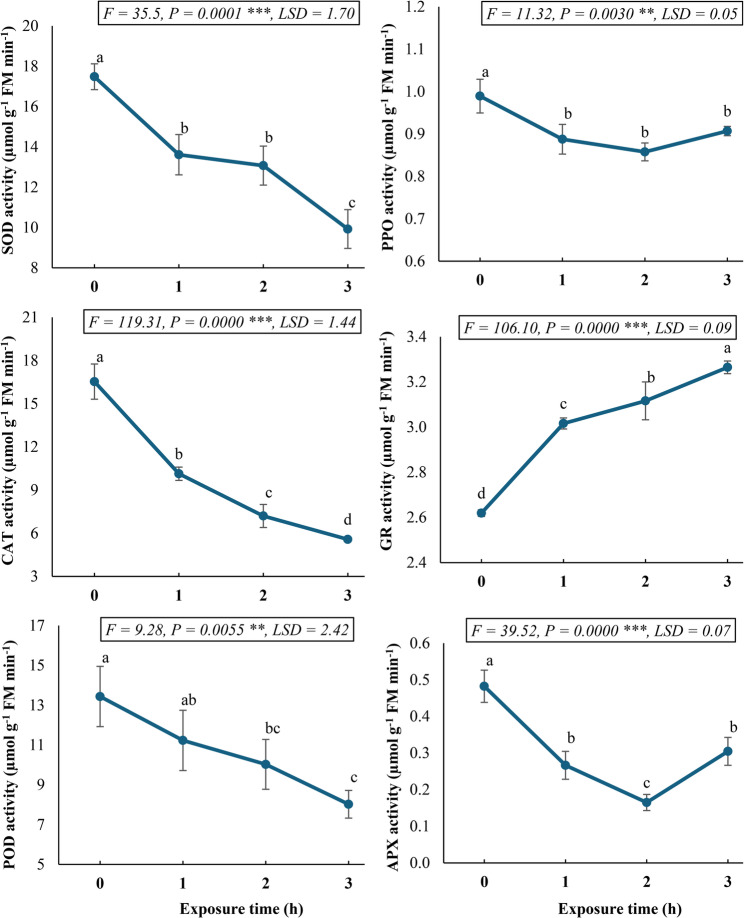
Antioxidant enzyme activities (SOD, CAT, POD, PPO, GR, and APX) in *Calotropis procera* callus culture following exposure to a 150 mT static magnetic field for varying durations. Different letters indicate statistically significant differences (*P* < 0.05) as determined by Duncan post-hoc test

Figure 5 illustrates the dynamic response of antioxidant enzyme systems to SMF exposure in *C. procera* callus cultures. The enzymatic antioxidants exhibited distinct temporal patterns, revealing complex regulation of the cellular oxidative defense machinery. Catalase (CAT), peroxidase (POD), and superoxide dismutase (SOD) activities demonstrated progressive downregulation with increasing SMF exposure duration. After 3 h of treatment, enzymatic activities were reduced to 0.34-, 0.59-, and 0.57-fold of control values, respectively, indicating significant (*P* < 0.05) deactivation of these primary antioxidant enzymes. The ascorbate-glutathione cycle components displayed more complex regulation. Ascorbate peroxidase (APX) activity showed biphasic modulation, with initial decline (minimum 0.30-fold of control at 2 h) followed by partial recovery at 3 h. Similarly, polyphenol oxidase (PPO) activity reached its nadir (0.13-fold reduction) after 2 h before showing marginal retrieval at the 3-h timepoint, though remaining significantly (*P* < 0.05) below control levels. In contrast, glutathione reductase (GR) exhibited sustained upregulation, achieving maximal activity (1.2-fold increase versus control) after 3 h of SMF exposure. This differential and coordinated temporal patterns of enzyme activities indicate the complexity of oxidative stress defenses under SMF exposure.

### Gene expression profile of phenylpropanoid pathway enzymes in *C. procera* under SMF exposure

**Fig. 6:**
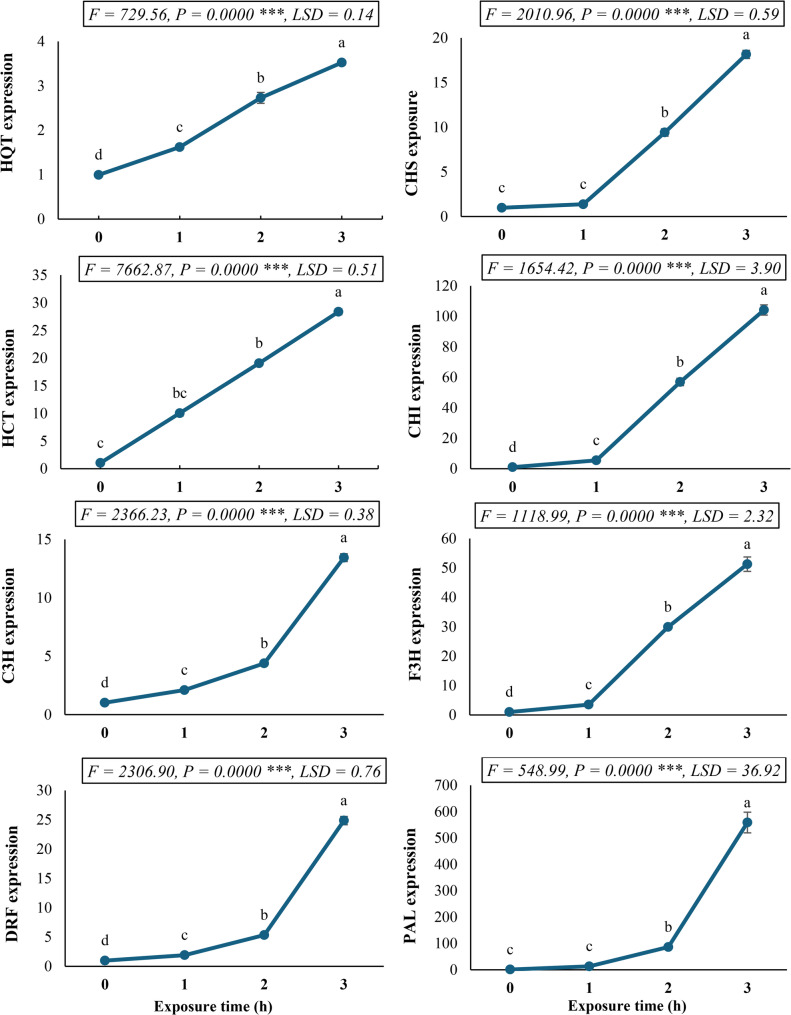
Gene expression profile of phenylpropanoid pathway enzymes (HQT, C3H, DRF, CHI, F3H, and PAL) in *Calotropis procera* callus culture following exposure to a 150 mT static magnetic field for varying durations. Different letters indicate statistically significant differences (*P* < 0.05) as determined by Duncan post-hoc test

Quantitative gene expression analysis revealed significant (*P* < 0.05) upregulation of all examined phenylpropanoid biosynthetic genes in *C. procera* callus cultures following SMF treatment (Fig. 6). The transcriptional response exhibited a consistent time-dependent enhancement pattern, with maximal induction observed after 3 h of SMF exposure. Notably, phenylalanine ammonia-lyase (PAL), the gateway enzyme of the pathway, demonstrated extraordinary 549.89-fold upregulation compared to untreated controls, suggesting profound activation of the phenylpropanoid flux under electromagnetic stimulation.

The investigated expression of the tracked enzymes displayed varying degrees of induction, with hydroxycinnamoyl-CoA: shikimate hydroxycinnamoyl transferase (HCT) and dihydroflavonol 4-reductase (DFR) showing 26.19-fold and 24.39-fold increases, respectively. Chalcone isomerase (CHI) and flavanone 3-hydroxylase (F3H) exhibited particularly strong responses, reaching 100.60-fold and 50.90-fold induction levels. The coordinated upregulation of hydroxycinnamate-CoA quinate hydroxycinnamoyl transferase (HQT; 2.54-fold), *p-*coumarate 3-hydroxylase (C3H; 12.20-fold), and chalcone synthase (CHS; 17.50-fold) completes the comprehensive transcriptional activation profile across the entire pathway. Accordingly, our findings demonstrate systematic magnetic induction of phenylpropanoid metabolism, with particularly remarkable effects on the early (PAL) and late (CHI, F3H) pathway components. The differential fold-change magnitudes suggest potential rate-limiting steps and branch point regulation in the SMF-mediated metabolic reprogramming of secondary metabolism in *C. procera*.

### Multivariate correlation analysis of SMF-induced metabolic and transcriptional responses in *C. procera* callus culture

**Fig. 7:**
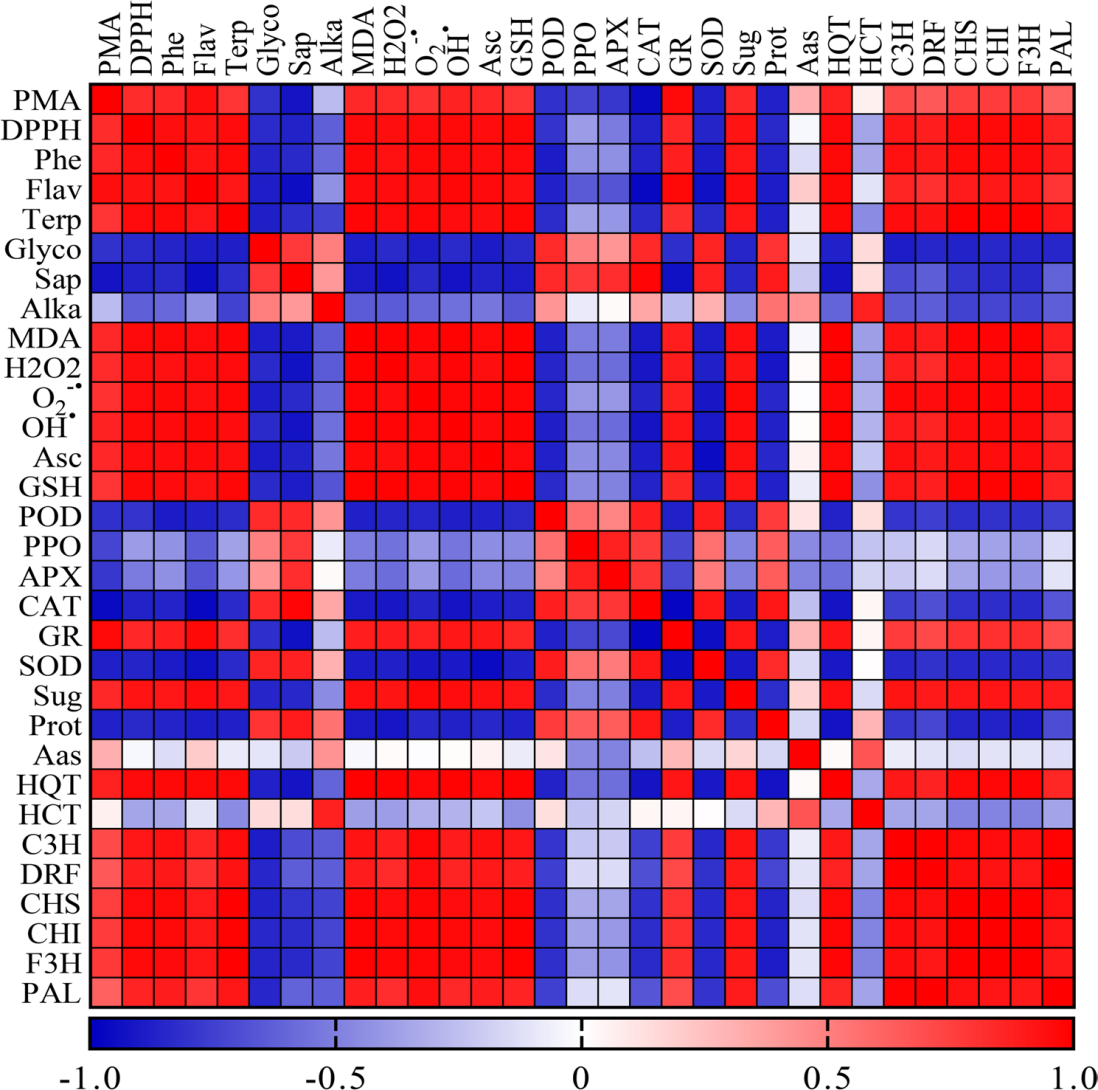
Pearson pairwise correlation heatmap among the assessed attributes in *Calotropis procera* callus exposed to a 150 mT static magnetic field for varying durations

The correlation between the studied physiological, biochemical, and genetic traits of *C. procera* callus culture exposed to SMF was evaluated using a correlation matrix heatmap, highlighting significant positive and negative associations among them (Fig. 7). A particularly strong positive correlation (*r* > 0.95) was observed among the oxidative stress markers MDA, H_2_O_2_, O_2_^−•^, and OH^•^, confirming their coordinated accumulation under SMF-induced stress. DDPH scavenging activity showed a highly significant (*r* > 0.90) positive correlation with phenols, flavonoids, ascorbic acid, and GSH contents, reinforcing the antioxidant roles of these compounds. Additionally, DDPH activity also correlated positively with expression levels of HQT, HCT, C3H, CHS, CHI, and F3H genes, suggesting the involvement of these genes in the enhanced production of antioxidant-rich secondary metabolites.

A notable positive correlation was found between terpenoid content and the expression levels of HQT, C3H, DRF, CHS, CHI, PAL, and F3H genes, implying a regulatory connection between phenolic biosynthetic gene expression and terpenoid accumulation. Also, a strong positive correlation was noticed between soluble sugar content and expression level of HQT, C3H, DRF, CHS, CHI, and F3H genes, suggesting the dual function of sugars as metabolic precursors and signaling molecules regulating phenolic and flavonoids biosynthesis. PAL expression levels showed a highly positive correlation with C3H, DRF, CHS, and CHI genes expression, reflecting their coordinated role in the phenylpropanoid and flavonoid biosynthetic pathway.

Furthermore, MDA, H_2_O_2_, O_2_^−•^, and OH^•^ collectively exhibited a strong negative correlation (*r* < -0.85) with the total antioxidant capacity (PMA) and the major non-enzymatic antioxidants, including phenols, flavonoids, ascorbic acid, and GSH, underscoring the critical role of this antioxidant system in countering SMF-induced oxidative damage. Soluble protein content was also negatively correlated with DPPH scavenging activity, total phenolic content, flavonoids, terpenoids, soluble sugars, and GSH, possibly indicating a stress-induced shift in metabolic allocation. Additionally, the activities of antioxidant enzymes CAT, POD, and SOD showed a negative correlation with the expression levels of key flavonoid biosynthetic genes, including CHS, CHI, F3H, and HQT. This inverse relationship may reflect a regulatory balance between enzymatic and biosynthetic antioxidant responses under SMF exposure. Furthermore, CAT, POD, and SOD activities were also negatively correlated with total antioxidant capacity (PMA), DPPH^•^ scavenging activity, and the accumulation of phenols, flavonoids, and terpenoids. Notably, a strong negative correlation was identified between the enzymatic antioxidants (CAT, POD, APX, SOD) and the non-enzymatic antioxidant glutathione (GSH), suggesting a potential compensatory relationship between these two defense systems. These patterns suggest that under SMF-induced stress, the *C. procera* callus may favor the accumulation of non-enzymatic antioxidants over the activation of enzymatic defenses.

## Discussion

The magnetic field stimulates cell growth and influences the biochemical composition of plants depending on factors such as the intensity and duration of exposure [41]. This study investigates the use of a static magnetic field (SMF) as a sustainable elicitor in *Calotropis procera* callus cultures. Given the potential of SMF to enhance phytochemical production, we assessed its effects on primary and secondary metabolite accumulation, antioxidant activity, and the transcriptional regulation of key phenylpropanoid pathway genes. Our study on *C. procera* callus cultures exposed to SMF revealed dynamic changes in primary metabolite accumulation, suggesting that SMF influences metabolic pathways in a time-dependent manner (Fig. 1). The study declared a progressive increase in total soluble sugars, peaking at 3 h post-SMF exposure with a 1.6-fold increase compared to untreated controls. This observed metabolic enhancement may be attributed to SMF-induced modulation of carbohydrate metabolism, potentially through stimulation of key enzymatic reactions in the Calvin-Benson cycle. Such an effect could enhance CO_2_ assimilation efficiency, thereby increasing photosynthetic carbon fixation and ultimately elevating soluble sugar accumulation [42].

In contrast to sugars, soluble protein levels decreased significantly, reaching a minimum (0.47-fold of control) after 3 h of SMF exposure, and free amino acid levels exhibited a biphasic response, with an initial 2.6-fold surge at 1 h, followed by a gradual decline, though remaining elevated (0.8-fold) after 3 h. This contrasting pattern may reflect a shift in the metabolic priorities of the plant under stress. In this case, the reduced amino acid levels might indicate their increased use in the production of stress-related compounds, including secondary metabolites, or their role in signaling pathways involved in the defense response of the plant. Similar responses have been documented in plants under osmotic or drought stress, where soluble sugar accumulation aids in osmotic adjustment while proteolysis releases amino acids to serve as osmolytes or substrates for stress-related metabolic pathways [43]. These findings also mirror previous observations under SMF exposure. Yao and Shen [44] reported that magnetic water treatment during the germination of *Tilia miqueliana* seeds led to a reduction in starch and protein levels, while increasing the content of reducing sugars. This decline may result from protein degradation due to oxidative stress or the activation of proteolytic enzymes under magnetic field exposure. Alternatively, SMF may redirect nitrogen metabolism toward amino acid synthesis rather than protein assembly, as suggested by the observed increase in free amino acids. Thereby, the transient increase in free amino acids suggests that SMF may stimulate amino acid biosynthesis pathways, possibly via enhanced nitrate reductase activity [45]. The subsequent decline could be due to their utilization in secondary metabolite synthesis or stress-responsive pathways. Accordingly, these metabolic shifts may reflect adaptive responses to SMF-induced stress, potentially optimizing energy allocation for growth and defense mechanisms.

The duration of static magnetic field (SMF) exposure significantly influenced secondary metabolite biosynthesis in *C. procera* callus cultures (Fig. 1), with distinct time-based accumulation patterns observed across different phytochemical classes. These metabolic shifts are particularly relevant given the therapeutic importance of *C. procera* secondary compounds. The most remarkable SMF effects occurred in phenylpropanoid metabolism, with flavonoids increasing 3.2-fold and phenolic compounds surging 7.5-fold after 3 h exposure. This aligns with findings by Wang et al. [12], who reported SMF-induced upregulation of phenylalanine ammonia-lyase (PAL) activity in quinoa, a key enzyme channeling carbon flux into phenylpropanoids. The particularly strong phenolic response suggests SMF may preferentially stimulate early shikimate pathway enzymes. These findings are consistent with earlier research on magneto-elicitation in plant tissue cultures, which indicated enhanced secondary metabolic content and antioxidant activity due to magnetic exposure [46]. *Silybum marianum* treated with 4 mT SMF led to increased phenolic and flavonoid accumulation [47].

Terpenoid accumulation showed more reasonable (1.6-fold) increases, consistent with Xu et al. [48], who observed similar SMF effects on terpenoid synthase in ginger. The differential response compared to phenylpropanoids implies branch-point regulation in precursor (acetyl-CoA) allocation. Contrasting patterns emerged for steroidal compounds, whereas cardiac glycosides progressively declined to 0.6-fold of controls, and saponins showed faster reduction (0.65-fold by 2 h). The decline in saponin and cardiac glycoside accumulation under SMF exposure can be attributed to several interconnected mechanisms. Primarily, SMFs may inhibit their biosynthetic pathways by interfering with the activity or gene expression of key enzymes, potentially through alterations in the cellular redox state or ion concentrations [49]. Concurrently, SMF-induced increases in hydrolytic enzyme activity or oxidative stress could promote the degradation of these glycosidic compounds. The progressive nature of the decline suggests a sustained SMF influence on the metabolic machinery, favoring pathways other than the biosynthesis of these steroidal compounds.

The observed biphasic alkaloid response in *C. procera*, characterized by an initial 0.61-fold increase at 1st h followed by a 0.34-fold decrease by 3 h, coupled with mirroring free amino acid fluctuations, suggests a dynamic interplay between stress perception, amino acid metabolism, and secondary metabolite synthesis. This phenomenon can be elucidated by considering the role of key enzymes like ornithine decarboxylase (ODC) and the intricate regulatory mechanisms governing nitrogen allocation in plants under stress conditions. The increase in alkaloid accumulation after 1 h of SMF exposure likely represents an acute stress response in *C. procera*. Plants often upregulate secondary metabolite synthesis, including alkaloids, as a defense mechanism against various stressors [50]. This rapid induction could be mediated by the activation of key enzymes in alkaloid biosynthesis pathways. So, the initial stress-induced activation of ornithine decarboxylase (ODC) could lead to a transient increase in the availability of precursors for alkaloid synthesis, resulting in the observed early increase [51]. However, the subsequent 0.34-fold decrease in alkaloid levels by 3 h suggests a shift in metabolic priorities. Under prolonged or severe stress, plants may reallocate resources, diverting free amino acid precursors away from secondary metabolism towards primary metabolic processes essential for survival, such as protein synthesis or osmolyte production [52]. The biphasic response could thus reflect a finely tuned adaptive strategy, where an immediate defensive surge is followed by a more conservative resource management under SMF.

The observed, time-dependent accumulation of phenolic and flavonoid constituents in *C. procera* callus cultures in response to a 150 mT SMF aligns with the established role of SMFs as effective abiotic elicitors. Elicitors are known to trigger defense responses in plant cells, often leading to the enhanced biosynthesis of secondary metabolites [53]. The coordinated upregulation of diverse compounds, from the highly abundant kaempferol and ellagic acid to less abundant constituents, suggests systemic activation of the biosynthetic machinery rather than the stimulation of a single pathway. The pronounced increase in specific compounds is particularly noteworthy. The 115% increase in kaempferol, a potent flavonol, is significant as flavonols are crucial antioxidants. Similarly, the doubling of ellagic acid, a polyphenol with well-documented radical-scavenging and anti-inflammatory properties, indicates a robust defensive activation [54]. The simultaneous rise in key phenolic acids like gallic, salicylic, and ferulic acid, which are integral components of the phenylpropanoid pathway, further supports this. The proposed mechanism for this phenomenon involves SMF-induced oxidative stress.

Studies of Raipuria et al. [55] and Saletnik et al. [41] have shown that SMF exposure can lead to the generation of ROS in plant tissues. This ROS burst acts as a signaling molecule that stimulates the activity of pivotal enzymes in the phenylpropanoid pathway, such as phenylalanine ammonia-lyase (PAL), which is a key gatekeeper enzyme channeling carbon from primary metabolism into the synthesis of phenolics and flavonoids [56]. The fact that all 18 identified compounds increased, regardless of their intial concentration or chemical class, strongly indicates that the SMF stimulus acts at a regulatory node upstream in the phenylpropanoid pathway. This holistic enhancement of the phytochemical profile demonstrates the efficacy of SMF as a sustainable physical elicitor for enhancing the production of valuable antioxidants in plant in vitro systems, without the drawbacks associated with chemical elicitors.

The exposure to a 150 mT SMF induced a significant and time-dependent oxidative stress response in *C. procera* callus cultures. The massive increase in O_2_^-•^ is a classic hallmark of abiotic stress in plant tissues. This primary ROS is often generated via the enhanced leakage of electrons to molecular oxygen from electron transport chains in chloroplasts and mitochondria [57]. The subsequent dismutation of O_2_^-•^, either spontaneously or via superoxide dismutase, leads to the formation of H_2_O_2_, whose levels also increased substantially.

The most damaging consequence of ROS cascade is the high increase in OH^•^, which is the most reactive and cytotoxic oxygen species. Its generation is typically mediated through metal-catalyzed reactions, such as the Fenton reaction, where H_2_O_2_ reacts with Fe_2_^+^ [58]. The OH^•^ radical attacks all major biomolecules, but its effect on membrane lipids is particularly destructive, leading to lipid peroxidation. This is directly evidenced by the profound increase in MDA, a well-established diagnostic marker for oxidative damage to cellular membranes. The strong correlation between the rising levels of ROS (O_2_^-•^, H_2_O_2_, and OH^•^) and the end-product of their activity (MDA) confirms a coherent and escalating oxidative event. Collectively, this coordinated upsurge in oxidative biomarkers confirms that the applied SMF acts as a potent abiotic elicitor. The data suggests that the SMF stimulus overwhelms the antioxidant defense capacity of the callus, leading to a condition of severe oxidative stress. This oxidative burst is a well-documented mechanism by which various physical stressors, including SMFs, trigger defense signaling pathways, which can subsequently lead to the activation of secondary metabolite biosynthesis as a protective measure [41].

The antioxidant capacity of plant tissues is intrinsically linked to their endogenous content of both enzymatic and non-enzymatic antioxidants, which collectively maintain cellular redox homeostasis and mitigate oxidative stress [59]. Our investigation on *C. procera* callus culture exposed to a SMF revealed a significant time-based modulation of key non-enzymatic antioxidants, ascorbic acid (AsA) and reduced glutathione (GSH). This observation aligns with existing literature indicating that SMFs can influence plant physiological processes, including their antioxidant defense systems [41, 60]. Our findings demonstrated a significant exposure time-dependent accumulation of both AsA and GSH, with maximal concentrations attained after 3 h of SMF treatment. Specifically, AsA content showed a 6.91% increase over the control value, while GSH showed a substantial 25.93% enhancement relative to the untreated culture control level. This differential magnitude of response between AsA and GSH suggests concurrent activation of their respective biosynthetic pathways or recycling mechanisms under SMF stimulation. The observed increases in these crucial non-enzymatic antioxidants are consistent with the understanding that plants often upregulate their antioxidant machinery as an adaptive response to various environmental stimuli, including magnetic fields, to counteract potential oxidative imbalances [61, 62].

Significantly, the upregulation of cellular antioxidants following SMF exposure in *C. procera* was directly correlated with an enhanced antioxidant potential. Both DPPH radical scavenging activity and total antioxidant activity, as measured by the phosphomolybdenum reduction assay, exhibited progressive enhancement with increasing SMF exposure duration. After 3 h of exposure, *C. procera* callus culture demonstrated a 2.32-fold greater DPPH radical scavenging activity and a 0.30-fold higher total antioxidant capacity compared to untreated control levels. This synchronous increase in antioxidant metabolites (AsA and GSH) and the corresponding antioxidant activity strongly suggests a coordinated activation of the cellular redox buffering system in response to SMF exposure. The findings underscore that SMF acts as a stimulus, triggering a time-dependent antioxidant response in *C. procera* callus cultures, thereby bolstering their capacity to neutralize reactive oxygen species and maintain cellular integrity [63].

The interplay of the antioxidant system and the level of endogenous ROS is a crucial modulator in sustaining plant survival under various environmental signals. Our results regarding *C. procera* callus culture exposure to a SMF revealed distinct and dynamic time-dependent patterns in the activities of key antioxidant enzymes, indicating a concomitant and complex regulation of the cellular oxidative defense machinery. Specifically, catalase (CAT), peroxidase (POD), and superoxide dismutase (SOD) activities demonstrated a progressive decline with increasing SMF exposure duration. After 3 h of exposure, their activities were significantly reduced to 0.34-, 0.59-, and 0.57-fold of the SMF-unexposed control activities, respectively. This decrease in the activities of principal antioxidant enzymes, which are crucial for detoxifying ROS like H_2_O_2_ and O_2_^•⁻^, suggests that SMF exposure might either directly inhibit these enzymes or alter the cellular signaling pathways that regulate their expression and activity [64]. Abdolmaleki et al. [65] reported that SMF can induce changes in plant enzyme activities, though the direction of change can diverge depending on the magnetic field parameters and plant species.

The components of the ascorbate-glutathione cycle, vital for sustaining the redox state of ascorbate and glutathione, exhibited more intricate regulation. Ascorbate peroxidase (APX) activity showed biphasic modulation, with a significant decline (0.30-fold of control activity after 2 h) followed by partial retrieval at 3 h exposure time. Likewise, polyphenol oxidase (PPO) activity reached its nadir (0.13-fold reduction) after 2 h before showing marginal retrieval at the 3-h timepoint, though remaining significantly below control levels. This biphasic response might indicate an initial stress-induced suppression, followed by a partial recovery as the cells adapt or activate alternative defense mechanisms. In contrast, glutathione reductase (GR) exhibited continuously increased activity, attaining maximal activity (1.2-fold increase compared to control) after 3 h of SMF exposure. This continuous increase in GR activity suggests a compensatory mechanism, where the cell prioritizes the regeneration of GSH to support other antioxidant processes, concomitant with the observed increase in GSH content [63]. The differential and coordinated time-dependent patterns of these enzyme activities underscore the complexity of oxidative stress defenses under SMF exposure. The decline of CAT, POD, and SOD, coupled with the multifaceted response of APX and PPO, and the continually increased activity of GR, collectively sustain dynamic rebalancing of the antioxidant status. This suggests that SMF exposure does not simply induce a general oxidative stress response but rather triggers a specific and time-dependent modulation of the antioxidant system in *C. procera* callus cultures, potentially shifting the reliance from certain primary ROS-scavenging enzymes to others, or emphasizing the role of non-enzymatic antioxidants and their regeneration [59].

The phenylpropanoid pathway is a pivotal secondary metabolic route in plants, responsible for synthesizing a vast array of compounds with diverse functions, including defense, pigmentation, and structural support [41]. Our quantitative gene expression analysis in *C. procera* callus culture exposed to SMF revealed a significant and coordinated upregulation of all examined phenylpropanoid biosynthetic genes. This transcriptional response exhibited a consistent time-dependent enhancement pattern, culminating in maximal induction after 3 h of SMF exposure, indicating a profound activation of the phenylpropanoid flux under SMF stimulation. Notably, phenylalanine ammonia-lyase (PAL), the gateway enzyme of the pathway, demonstrated an astonishing 549.89-fold upregulation compared to the untreated control. This dramatic increase in PAL expression signifies a robust initiation of phenylpropanoid metabolism, channeling primary metabolites into this secondary pathway. The investigated expression of other tracked enzymes also displayed varying degrees of induction, highlighting a comprehensive activation across different branches of the pathway. Hydroxycinnamoyl-CoA: shikimate hydroxycinnamoyl transferase (HCT) and dihydroflavonol 4-reductase (DFR) showed 26.19- and 24.39-fold increases, respectively. Chalcone isomerase (CHI) and flavanone 3-hydroxylase (F3H) exhibited predominantly strong responses, reaching 100.60- and 50.90-fold induction levels, respectively. The coordinated upregulation of hydroxycinnamate-CoA quinate hydroxycinnamoyl transferase (HQT; 2.54-fold), *p*-coumarate 3-hydroxylase (C3H; 12.20-fold), and chalcone synthase (CHS; 17.50-fold) further completes this comprehensive transcriptional activation profile across the entire pathway [60].

Our findings demonstrated that SMF exposure triggered the induction of phenylpropanoid metabolism in *C. procera* callus cultures. The remarkable effects on the early (PAL) and late (CHI, F3H) pathway components suggest potential rate-limiting steps and branch point regulation in the SMF-mediated metabolic reprogramming of secondary metabolism. The differential fold-change magnitudes across the pathway enzymes indicate that SMF acts as a potent elicitor, composing a complex transcriptional cascade that prioritizes the synthesis of specific phenylpropanoid derivatives. This metabolic reprogramming could be a stress-adaptive response, as many phenylpropanoids are known to play crucial roles in plant defense against various environmental challenges [66, 67]. The observed upregulation suggests that *C. procera* might be enhancing its protective mechanisms in response to the SMF stimulus, leading to an increased capacity for producing these significant secondary metabolites, reflecting the typical plant response to mild abiotic stressors.

## Conclusion

The biochemical and molecular analyses of *C. procera* callus cultures subjected to various time intervals (0–3 h) static magnetic field (SMF) revealed a multifaceted biochemical and molecular response characterized by significant metabolic reprogramming. SMF acted as a potent elicitor, initiating a rigorous oxidative burst evidenced by the coordinated accumulation of MDA, H_2_O_2_, O_2_^-•^, and OH^•^, which in turn triggered time-dependent shifts in primary and secondary metabolism, enhancing phenylpropanoid biosynthesis while suppressing steroidal compounds accumulation. The pronounced upregulation of phenylpropanoid pathway genes, particularly PAL, underscores the activation of defense-related metabolic routes. This was further reflected in the specific, coordinated upregulation of 18 phenolic and flavonoid compounds, including kaempferol and ellagic acid. Concurrently, the enhancement of non-enzymatic antioxidants (ascorbic acid and glutathione) and total antioxidant capacity indicates an adaptive oxidative stress response. The decline in enzymatic antioxidant activities (CAT, POD, SOD) alongside their strong negative correlation with non-enzymatic defenses suggests a strategic reallocation of cellular resources toward biosynthetic rather than scavenging pathways under SMF-induced stress. Correlation analyses revealed a synergistic relationship between phenolic accumulation and antioxidant activity, and a strong inverse relationship between oxidative stress markers and the antioxidant system. These findings position SMF as a sustainable, non-invasive biophysical approach for triggering pharmaceutical phytochemicals in plant cell cultures. Further research should focus on optimizing SMF parameters and exploring transcriptomic and proteomic changes to fully harness its potential for metabolic engineering for future applications in agricultural biotechnology and pharmaceutical production.

## Supplementary Information

Supplementary Material 1.

## Data Availability

The datasets used and/or analysed during the current study are available from the corresponding author on reasonable request.
