# Supplementary material for: Static magnetic field-induced metabolic shifting: coordinated phenylpropanoid induction and antioxidant system regulation in Calotropis procera callus culture
Source: BMC Plant Biol. 2026 Feb 11;26:376. doi: 10.1186/s12870-025-07997-3 (PMC12930588; doi:10.1186/s12870-025-07997-3)
Supplement: Supplementary file 1 — Supplementary Material 1. [file 12870_2025_7997_MOESM1_ESM.docx]

| 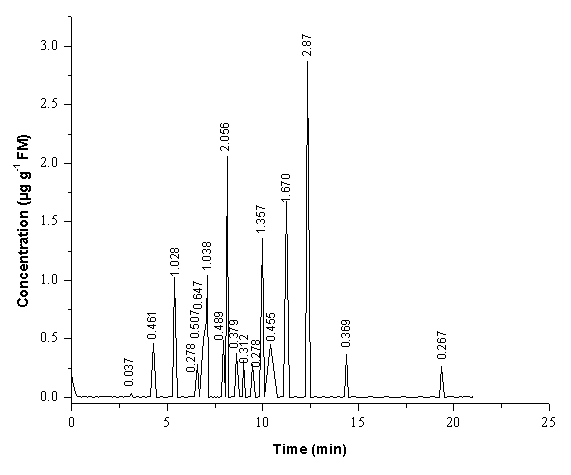  a | 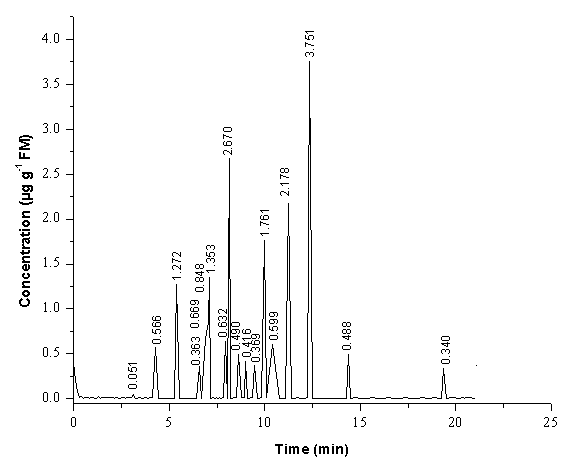  b |
| --- | --- |
| 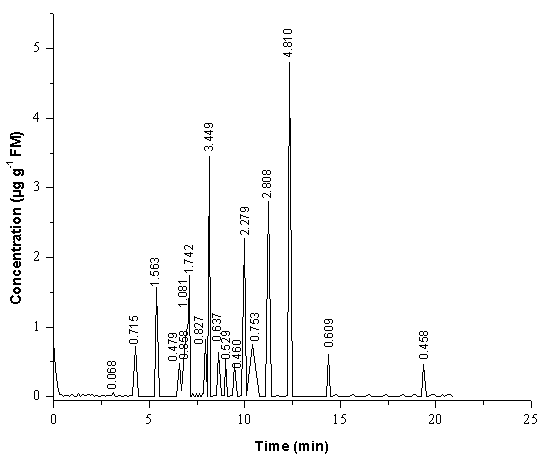  c | 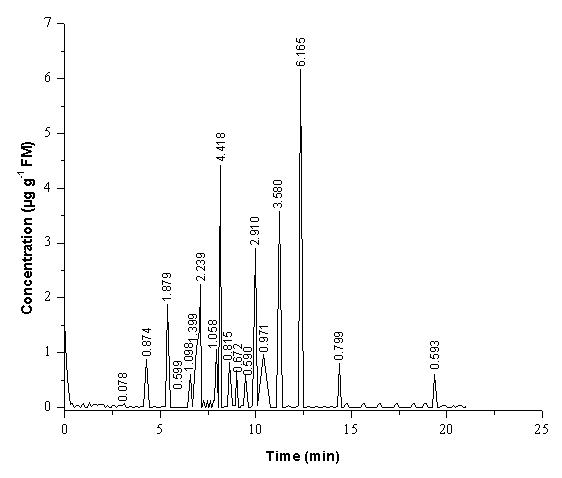  d |

Figure S1. HPLC chromatograms of phenolic and flavonoid compounds identified in *Calotropis procera* callus cultures. Profiles are shown for callus cultures exposed to a 150 mT static magnetic field for different time intervals: (a) 0h, (b) 1h, (c) 2h, and (d) 3h.
